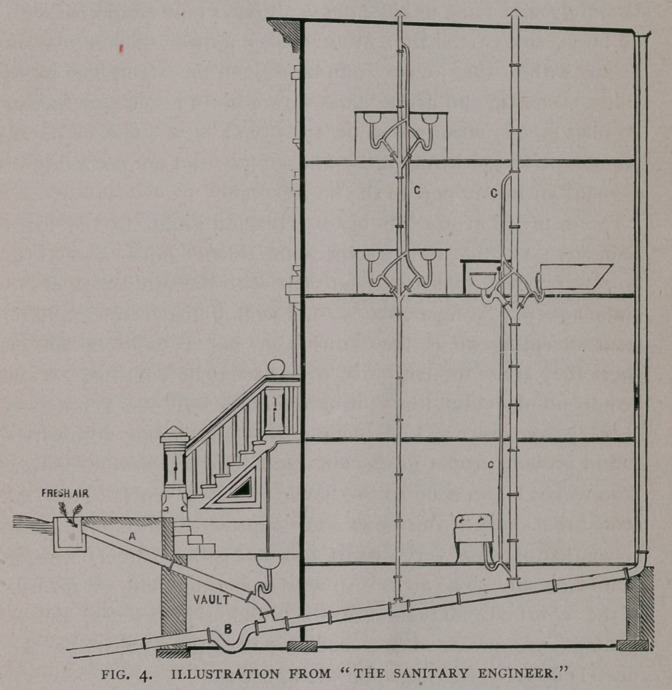# “Sewer Gas” and Its Dangers

**Published:** 1882-07

**Authors:** A. R. Davidson


### "Sewer Gas" and its Dangers.*

By A. R. Davidson, M. D.

At the last meeting of this association one of our members reported the death, from diphtheria, of three children in one family resident upon Delaware avenue. Subsequently it was discovered that the drainage of the house was defective and ready admission afforded to sewer air. The same physician reported three cases of diphtheria in another family, resident in a dwelling supplied with the recent improvements in drainage, and certainly free from all suspicion of sewer air contamination. In this last case the children all recovered. The discussion which

*Read before the Buffalo Medical and Surgical Association, July 1st, 1882.

followed showed some divergence of opinion among ourselves as to the dangers arising from sewer air; but the chief incentive which has induced me to offer a paper upon the subject, is the publicity which the sad cases, reported by Dr. Rochester, have received, and the public alarm and uneasiness created by over-talking and sensational statements concerning sewer gas, and its effects upon the health of the community.

One widely circulated newspaper asserts "on the authority of our most eminent physicians" that "diphtheria is caused by a subtle and insidious gas—the more terrible because it can not always be detected by the smell." A plumber, in the columns of the same paper, recites at length his experience of the noxious qualities of the gas, and gives to the public and profession new light as to the ætiology of diphtheria and other diseases by the statement that "from imperfect sewers escape *little brown bugs* invisible to the eye, which lodge in the throat, causing a bronchial affection—in time they originate disease and then death." Such extraordinary statements are by no means confined to our own city. A San Francisco paper for instance, heads an article "*Died* of *Sewer Gas*, 658," as we read further however, we find that the editor has attributed all the deaths in excess of those for the same period in the preceeding year to its deadly influence, and claims to be supported in this assertion by "the Board of Health and all the best physicians of the city."

The happy householder who has enjoyed a blissful feeling of security concerning the sanitary condition of his house, is thus suddenly awakened to a sense of hazard and possible danger to his own little ones. He asks the advice of a number of persons and finds that in a multitude of counselors there is confusion. One man tells him that his fixtures are all right. Another that his traps are useless. If he ventures the hope that they must be effective, as he has never perceived any smell, he is informed that "pure sewer gas has no odor and is consequently the more deadly the less odorous it is." So from one plumber he receives hope and by another he is plunged into dispair, and finally, in a state of ludricrous bewilderment, he usually appeals to his family physician; unfortunately there is no lack of contradictory opinions and recommendations from medical men; there is hardly an ill of the human body and scarcely one of the human mind which has not been by some attributed to the malignant and subtle influences of sewer gas.

In fact, however, as I stated in the discussion last month, there is no such thing as sewer gas. Sewers contain air, mixed with various kinds of gases, in greatly varying proportions. These gases do not and can not produce specific contagious diseases, such as diphtheria, scarlet fever or typhoid fever. Our own State Board of Health officially says: "*No such gas as sewer gas exists and there is absolutely no proof that the diseases which attend the admission of sewer air into our dwellings, are produced by gases.*"

The term "sewer gas" is often used by chemists and sanitarians, but only as synonomous with sewer air, and this has led to the popular error that sewer gas is a distinct gaseous body, having peculiar and marked characteristics of its own.

The composition of sewer air is ordinary air, with the addition of rarely over half per cent. of carbonic acid, one per cent. of sulphuretted hydrogen and traces of ammonia and fœrtid organic matter. The very common belief that sewer air is, in itself, particularly noxious, is a mistake. The mortality among the men who work in sewers, sometimes for hours daily, is no greater than that of the same class of laborers engaged in other avocations. If "sewer gas" was as deadly as reported, the mortality would indeed be frightful, for I venture to say that of the houses of this city connected with the sewerage system, nine-tenths are more or le. s accessible to it.

In the great majority of cases sewer air is not even dangerous, except it be taken in very concentrated doses, or breathed over and over again in a living room. In the later case I believe that, though it cannot produce specific disease, it tends to produce headache, loss of appetite and a condition of debility (especially in children) which disposes the system to be more readily affected by the immediate and specific cause of disease. The connection, however, between diphtheria (for example) and bad plumbing is by no means that which many people seem to suppose, and the spread of diphtheria in houses which have no communication with the sewer is neither uncommon nor unaccountable. The disease, undoubtedly contagious, is propagated by micrococci coming from those affected by it. Sewer air is a possible means of conveying them after they have been introduced into the sewer with the discharges of the sick. It is by no means the only medium, and important as it is to exclude sewer air from our houses; it is not true that this exclusion forms an absolute safeguard against this or any other disease. The conditions existing in a well constructed sewer are not (though the contrary opinion is generally sustained) very favorable to the culture or preservation of the microbes of disease; and even if the sewers are not clean the danger arising from them may be very easily exaggerated; the sediment is mainly inorganic, and non-putrescible matter such as mud from the street and sand and gravel from roofs; the organic matter being lighter than water generally passes off, even in those in whtch there is a good deal of deposit. There is no doubt, however, that especially in large sewers, where the flow occupies but a small part of the arch, that the presence of ammonia with moisture and warmth would furnish an opportunity for the development and propagation of such contagia. And we may reasonably suppose that these bacteria or germs may become detached and be carried by currents into the outer air or through defective pipes into our dwellings. The possibility of this affords sufficient reason for the employment of the utmost care in the exclusion of sewer air from our houses. The fact that on 364 days in the year a little sewer air may enter without any injury to the inmates—is little comfort, when we realize, that we know not when the odd day may come, when it may bring with it the germs of disease.

*This possibility, however, is completely removed if communication between the street sewer and the house drain be cut off by a simple water trap and proper ventilation.* "The trap" then is an exceedingly important appliance, and as there are many physicians who are not familiar with those in use by plumbers, I do not perhaps need to apologize for calling your attention to some common forms of them. Of patent traps there are no end; each claiming on its honor as a trap to be impervious to sewer air. I will call your attention to the two of them most highly commended by good authorities. Both are a combination of a water seal and a mechanical obstruction, and for use under sinks, bath tubs, wash bowls, &c., they offer some advantages over the ordinary water traps.

Fig. 1.

Fig. 1, (see cut) is the Bower Trap. The inlet pipe A connecting directly with washstand, sink or other fixtures. B represents the outlet pipe, connecting directly with the main waste pipe. The cup-shaped chamber C remains filled with water to the level of the outlet pipe, thereby floating the hollow ball D firmly to the mouth of the pipe A, making a perfect seal.

Fig. 2.

Fig. 2 represents the Cudell Trap, also simple in its construction and not liable to get out of order. The ball, being made of metal, in this case closes the inlet pipe by its own gravitation. This trap offers one advantage over the first named in that its seal is good even in the absence of water. The patent traps are, of course, more expensive than the old fashioned S. trap, various forms of which I show you. (Fig. 3.)

Fig. 3.

No. I is what is known as the full S. No. 2 ¾ S., No. 3 ½ S., while Nos. 4, and 7 are respectively named the Running—and the Bag trap.

There is for ordinary use no better trap than the common S, properly ventilated. The objection is made to them with some reason, that when rooms are left unoccupied for some time the constant current of air passing through the vent pipe will cause the evaporation of the water and thereby unseal the trap. This may certainly happen, in hot weather especially. It is always a wise precaution when at hotels, for instance, on being shown into a room provided with a stationary wash stand, to at once turn a small stream of water from the faucet into the bowl, thus ensuring the sealing of the trap and security against any further admission of the sewer air.

But those of you who are familiar with the statements of persons interested in the sale of appliances alleged to be sewer gas excluders, or of chemicals possessed of magical disinfecting properties, or who have read the oft quoted article of Prof. F. H. Hamilton, read before the New York Academy of Medicine, entitled "The Struggle for Life against Civilization and Æstheticism," will perhaps reply; "the water trap is no security."

In the paper referred to Dr. Hamilton says: "The foul gases readily pass through water, and there is no security against sewer gas, but the proper use of chemicals, supplied to the traps daily"; perhaps recognizing the impossibility of carrying out such a recommendation, he further advises as the only real security, the placing of all plumbing fixtures in an annex to the building. Dr. Hamilton's views were seconded by Prof. Doremus, who advised that chemicals should be applied to the water traps every time they were used by a mechanical contrivance. He demonstrated the passage of sewer gases, ammonia and sulphuretted hydrogen through ½-in. glass traps, as I do now. (Experiment shown.) It will be seen that the proper reaction upon the test paper placed above the water in the trap, takes place in the case of the ammonia in 15 minutes with sulphuretted hydrogen in three to four hours. Those who have been alarmed by such statements, will take comfort from the sensible remarks of Dr. Billings, who said, referring to Prof. Doremus experiments, "that they did not prove anything as regards the passage of sewer gases through traps of water. The gases in the flasks are almost pure and the amount of water in the glass tubes very small and soon saturated. In the soil pipes the offensive gases are greatly diluted with air and the very careful experiments of Dr. Carmichael, of Glasgow, made with an ordinary water closet and a soil pipe, which had been used for a long time, showed conclusively that the amount of gases which pass through a water trap from a ventilated soil pipe, is so extremely minute that it can only be detected by the most delicate tests." The experiments referred to by Dr. Billings are published in the March number of the *Sanitary Journal*, of Glasgow, and were most carefully performed. The amount of ammonia which passed through an ordinary S trap by absorption on the surface of the water on one side and diffusion upon the other, varied from about 1-400 to 1-200 of a grain in 24 hours. Sulphuretted hydrogen passed through the trap in 24 hours to the extent of 1-100 of a grain, and carbonic acid 7 to 10½ grains in the same time. These are the quantities of the only sewerage gases existing in the soil pipe in estimable quantities, which pass through in an ordinary ventilated water closet trap in 24 hours. If the trap is not ventilated the amount is slightly increased. The result being ammonia 1-100 of a grain, sulphuretted hydrogen 1-60 of a grain, carbonic acid 32 grains, diffused into the atmosphere of a house gradually during the day; it need scarcely be said these quantities are absolutely harmless.

But the alarmist replies, granting that these experiments are conclusive, "the water trap is no protection against the germs of disease and if they pass over in any quantity, they will reproduce the disease." It is true that even the extreme dilution does not absolutely remove this danger, but the experiments of Dr. Carmichael show conclusively that germs, putrifactive or specific, will not p^ss through a sound water trap. Time will not allow me to recite the experiments referred to, but they prove conclusively the fact stated.* Prof. Janeway, of New York, and other competent observers make the same statement.

Water traps are therefore for the purpose for which they are employed perfectly trustworthy. They exclude the sewer air to such an extent that what escapes through the water is so little in amount and so purified by filtration as to be perfectly harmless. And they exclude entirely all germs and particles including the specific germs or contagia of disease.

We have them at our command a simple, complete safeguard against the evils conveyed by sewer air, but unfortunately I have also to add that through carelessness and ignorance we very seldom avail ourselves of it. Until late years, at least, traps have been so placed that in the great majority of cases they failed utterly to accomplish their purpose.

The not unusual course of the plumber is to tap the sewer, lay the drain pipe right into the dwelling, run up a stack of 4-in. soil pipe to second floor, place a full S trap right on top of vertical pipe, set both tub, wash stand, water closet, sink, &c., and report the jcb thoroughly done and everything safe. The householder is shown a trap upon the pipe leading to every fixture, and he is confident that there is no possibility of sewer air entering his house. The fact is, however, that such an arrangement is no protection at all. Each one of these traps are liable to be syphoned, and as a matter of fact may be unsealed most of the time. Again, as in our sewers we have no

---

* A reprint of Dr. Carmichael's article will be found in the *Sanitary Engineer* of New York, vol. III, page 212.

provision for ventilation, the gases having free access to the house, and in winter, especially, being drawn into it by the comparatively high temperature of our rooms will force the seals even when the water is in the trap. I am informed that of late years such plumbing is not often done, but I venture to say that half the houses of Buffalo have a drainage arrangement similar to this or one equally faulty.

Syphonage of the trap results from a vacuum, or partial vacuum, being formed in the waste pipe below the trap. When water enters the soil pipe, it displaces a body of air in front of it, forcing it either into the main sewer—the open air through ventilators, or into the house through weak water seals—at the same time the partial vacuum produced above empties the water out of every trap which it passes. If a perfect vacuum could be produced, the pressure of air would equal about 15 lbs. to the square inch; the column of water in a trap rarely exceeds 1½ inches, and it therefore would not require more than 1-240 part of a perfect vacuum to displace all the water on the inlet portions of a trap, so that air can pass through. This possibility of syphonage may be obviated by suitable ventilation of every trap, but we cannot have this without suitable ventilation of the whole house drain, and this is a matter of the utmost importance. To accomplish this, it is first necessary to carry the soil pipe through the house, giving it a ventilation above the roof. To this pipe *each trap* should be connected by a good-sized air pipe, having connection to it above all fixtures. We thus prevent the possibility of syphonage or the accumulation of gases in any pipe.

We have now provided an excellent ventilation for the sewers *through* our house. This is certainly a great improvement upon the first plan which ventilates the sewer by allowing the gases to flow *into* our house. I am informed a goodly number of our dwellings have gotten this far in the march of sanitary improvement. But with the possibility of germs of disease existing in the public sewers it is unwise to permit them to have ready access to the drainage system of our houses. Plumbing wears out, traps become syphoned or lose their seals by evaporation and we run risk enough in taking the chances of being poisoned by our own house drains without adding a thousand-fold to it, by having the foul air of the sewers sweeping through our drain. The words of Prof. Jenkin, of Edinburgh, are pertinent in this connection, though a little overcolored. "All town sewers must be treated as tainted, since, at some time or another the taint is sure to arise. True, the germ may get into our house drains, and breed millions of other germs, and yet we may escape, for our internal fittings may be so perfect as wholly to exclude the hostile army, but who would be so foolish as in a dare-devil way, to allow this army to lurk at every closet, at every sink, at every bath, in every pipe of our house waiting at a hundred outlets for just one opportunity to creep through, *when we could bar the door effectually at one main entrance.* To admit gas from the common sewer into the private drainage system of our house is to lay on poisoned air all over the house with taps to draw it off, placed at random, with the hope that by no accident no single tap may ever be turned the wrong way. Surely, one would think such folly could never be committed, yet not only is the practice common, being due to ignorance, but actually some modern sanitary engineers recommend arrangements by which every house is directly connected to the common sewer, trusting to the hundred little devices inside the dwelling to exclude the poison." In *Buck's Hygiene and Public Health* it is stated, "a common plan of arranging house drains is to carry the pipe directly into the sewer, without a break in the continuity and without providing a means of ventilation, dependence being placed upon the small traps in the house to exclude the sewer gas. This is a most delusive practice and cannot be too severely condemned." An efficient trap should be placed in the main drain just outside the house, but this plan is not complete, for it does not insure a free circulation of air through the house drains. An opening should be made in the drain pipe between the trap and the house, communicating with the outside air and protected above the surface of the ground by a grating, so as to form an air disconnection between the house pipe and the drain leading into the sew[illegible] To make this plain, I have here a drawing (see fig. 4,) which shows a house drain arranged as it should be, with a running trap in the main drain, and a ventilated pipe attached to the house side of the drain.

FIG. 4. ILLUSTRATION FROM "THE SANITARY ENGINEER."

Though the isolation of each house is so easily accomplished in the manner described, it is but rarely done. There is no novelty in the principle. It is commended by the highest sanitary authorities; properly done it completely cuts off all danger from sewer air, except that which is generated within the house, and which will not be poisonous except when contagious illness is present there. It is true that when there is much pressure of air in the sewers the trap may be forced, and to prevent this it is recommended to carry a pipe up to the roof from the sewer side of the trap; this completely does away with the possibility of the seal being broken; but if the sewers themselves are ventilated there is no need of this. It is, however, absolutely necessary to have the ventilating pipe connected with the house side of the trap, otherwise we impede the circulation of air within the house fixtures. Such a circulation is of course essential, and as the temperature in the soil pipe and in the inlet pipe is never the same, there will be a constant circulation of fresh air through the house pipes and no possibility of stagnant air in any part of the house drain.

The principal arguments against placing a trap in the main drain, are: 1st, that it may impede the flow of house waste into the sewers; 2nd, that it may interfere with the ventilation of the public sewers. As regards the first objection, that is not likely to occur excepting where the drains have not a sufficient fall, or where they are of too large size, or not adequately flushed. As to the second objection, that it may interfere with the ventilation of the public sewers, I would say that the public authorities should provide proper means for ventilating the sewers so that there would be no need to use house drains as vents. I am informed that most of our sewers are unprovided with any means for ventilation, thus very greatly increasing the dangers arising from sewer air. The air is displaced sometimes with great force by the constant fluctuation of the volume of sewage; unless openings are provided to accommodate these changes, the effect is directly exerted upon the house trap.

Mr. Latham, in Buck's *Hygiene*, recommends the method of ventilation by man-holes in the centre of the street, the openings should be large and sufficiently numerous to ensure complete ventilation of every part of the sewer. In no case in which houses are connected with the sewers, should the distance between ventilators be more than 200 yards. These openings should always be placed in the middle of the street, so that the escaping air shall be diluted as much as possible before reaching the sidewalk. Thorough dilution is the great safe-guard. If this be secured, sewer air is robbed of its noxiousness. Many of the English cities have introduced ventilation in the main sewers in the manner proposed. In the wide streets the foul gases mix so rapidly with the air that no unpleasant odors are perceived and the death-rate is said to have markedly lessened since the introduction of the thorough ventilation of the sewers.

There is much to be said on this and on other parts of my subject, of which time will not allow me to speak. It has been my object to bring to your attention simply the leading principles of house drainage, which have received the commendation of the best authorities. With the manifold details—in the choice of materials, of fittings, and of modes of designing and executing work—the physician has little to do. When consulted, however, he should insist on the five cardinal principles to which I have referred, viz:

1. Complete disconnection of house drain and street sewer.

2. Ventilating pipe at foot of house drain.

3. Soil pipe to be carried above the roof and not to be used as a conductor of rain water.*

4. Every fixture to be provided with a trap, suitably ventilated.

5. The whole system of piping inside to be water-tight and gas-tight.

In the execution of the work we are generally at the mercy of the plumber, and an inspection of our houses would show a great deal of careless and ignorant plumbing. The law provides some security for the favored citizens of New York and Brooklyn, by requiring the registration of every plumber and the submission of plans and inspection of all new work in dwellings, by the Board of Health. The benefits of this law should be ex-

*There is no objection to, but rather an advantage in the access of rain water to the house drains by an independent pipe, as shown in Fig. 4.

tended to Buffalo. The only people whom we would suppose would oppose it would be builders of pasteboard houses, and plumbers who do cheap and dangerous work. No honorable and self-respecting plumber can compete with men who lack both skill and conscience, and hence the latter class have full sway in cheap contract work.

The respectable plumbers of Buffalo have wealth and influence enough to procure the extension of this law to this city, and it is a matter of surprise that they have hitherto taken no action in the matter.

## Figures and Tables

**Fig. 1. f1:**
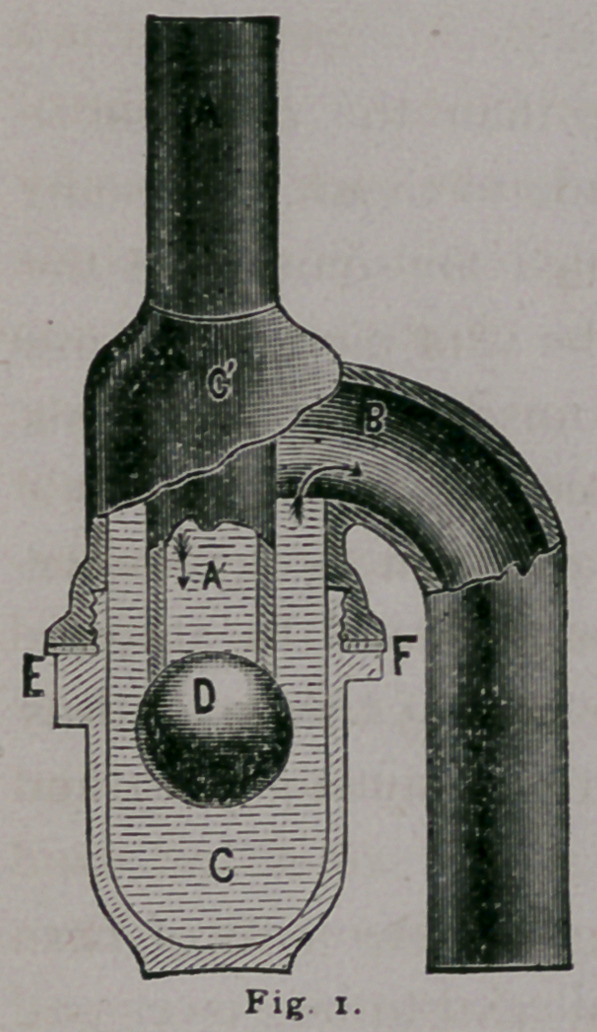


**Fig. 2. f2:**
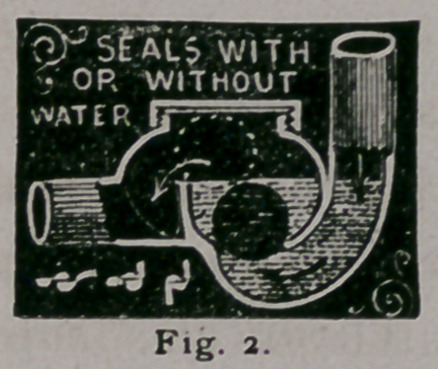


**Fig. 3. f3:**
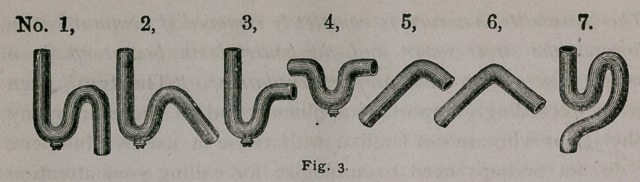


**Fig. 4. f4:**